# Distribution of hemoglobin and prevalence of anemia in 10 ethnic minorities in China: A population-based, cross-sectional study

**DOI:** 10.1097/MD.0000000000009286

**Published:** 2017-12-15

**Authors:** Xiuli Zhang, Yuan He, Xiaoxu Xie, Mengmeng Ji, Xu Ma, Zengli Yu

**Affiliations:** a Public Health College of Zhengzhou University, Zhengzhou; b National Research Institute for Family Planning; c Research Center for Population Health and Risk Assessment, National Human Genetic Resources Center; d Peking Union Medical College, Dongcheng District, Beijing, China.

**Keywords:** anemia, ethnic minorities, hemoglobin, women of child-bearing age

## Abstract

Racial differences have been reported in hemoglobin (Hb). However, distributions of Hb and anemia rate in ethnic minorities were rarely reported.

We aimed to observe whether there are ethnic differences in Hb distributions and prevalence of anemia.

The data included 480,699 women 20 to 49 years’ old from 10 ethnic minorities in China in 2014. Analyses of variance were used to examine the differences of Hb distribution among the 10 ethnic groups, as well as the differences in Hb level between different ages, education levels, occupations, and non- or ethnic enclaves in each ethnic group. *χ*2-test was adopted to analyze the differences in anemia rate among the 10 ethnic groups and between different ages and nonethnic or ethnic enclaves in each ethnic group.

The ethnic differences of the Hb distribution and anemia prevalence were observed in the 10 ethnic groups. The lowest mean Hb concentration was shown in Chuang (126.8 g/L), and the highest mean Hb concentration was in Tibetan (138.5 g/L). According to the World Health Organization criteria to define anemia, the highest prevalence was in Tibetan (46.9%) after the adjustment of Hb concentration for altitude, and the lowest prevalence was in Yi (10.6%). Furthermore, there were differences on mean Hb concentration or anemia rate in participants between ethnic enclaves and non-ethnic enclaves in most ethnic groups.

The ethnic differences of the Hb distribution and anemia prevalence were observed in the 10 ethnic groups, which might be associated with geographic conditions, genetic background, and eating habits.

## 1 Introduction

Hemoglobin (Hb) concentration is a critical indicator of anemia, and normal Hb distributions vary with age, sex, life style, race/ethnic, socioeconomic status, regional difference, and so on.^[1]^ Especially, ethnic groups have their own unique habits, geographic conditions, and human genetics, which may have significant effects on Hb concentrations. Previous studies showed that the racial differences of the Hb distribution have been recognized between African Americans and whites.^[2,3]^ In addition, for the populations in which the rate of inherited hemoglobinopathies was high, the mean Hb concentration might be lowered.^[1]^

According to the World Health Organization (WHO), anemia is defined as Hb concentration <120 g/L in nonpregnant women. Women of childbearing age are more susceptible to anemia because of the experience of menstruation, pregnancy, childbirth, and breastfeeding. Global anemia prevalence in nonpregnant women was 30% (1993–2005) according to the WHO. In the poverty-ridden Africa, the rate of anemia was the highest in nonpregnant, as 47.5%, and in the developed Europe, the prevalence reached 19% in nonpregnant women.^[1]^ In china, the women of childbearing age were also at a high risk for anemia and had a 15.4% prevalence of anemia.^[4]^

The People's Republic of China consists of Han nationality and 55 ethnic minorities. The ethnic groups mostly live in the remote areas with distinct geographical environmental and climates, and are commonly in poor socioeconomic status and with lower education level. Furthermore, they have their own unique eating habits and genetic backgrounds. For example, the Chuang likes rice wine and has high incidence of α- and β-thalassemia gene (26.9% and 19.9%, respectively).^[5]^ The minorities of Uygur, Hmong, Tujia, Yi, Buyi, and Hui live in the western China and their eating habits are also very different; for example, the Hmong favoring hot and spicy, Uygur and Tujia people drinking tea, and the Yi's diet rich in vegetable and lamb. Mongolian and Manchu, living in northeastern China, are lack of fresh vegetables and fruit because of the cold climate. Whereas the Tibetan people live in the Plateau with low barometric pressure, which could cause high-altitude hypoxia or decreased oxygen levels and like milk tea. Anyway these differences in ethnic groups might affect Hb distributions and the prevalence of anemia. However, few researches might provide valuable information on the status of the Hb distributions and the prevalence of anemia among ethnic groups in China.

Population-based measures of Hb are important for surveillance of anemia and the Hb distribution. Therefore, a nationwide, population-based, cross-sectional study was conducted to estimate the prevalence of anemia and observe whether there are ethnic differences in Hb distributions.

### 1.1 Population and study design

We performed a nationwide, population-based, cross-sectional study and obtained data about the Hb concentration and the prevalence of anemia from 10 minorities in china in 2014. The participants were from the National Free Preconception Health Examination Project (NFPHEP). The NFPHEP, which was supported by the Chinese National Health and Family Planning Commission and Ministry of Finance since 2010, aimed to provide free preconception health examinations for rural reproductive couples who planned to be pregnant within the next 6 months. All participants were established family health records by being provided health checkup, laboratory examination, and a standard questionnaire. Detailed information on data collection, protocols, and study design have been published elsewhere.^[6]^ A written informed consent form in Chinese was obtained from each participant before participation. The study was approved by the Institutional Research Review Board at the National Health and Family Planning Commission.

### 1.2 Procedures

A standard questionnaire was used to collect the baseline information of the participants by trained staff through face-to-face interviews in the local family planning service agencies. The basic information included age, education level, occupation, National origin, residence address, lifestyle (self-reported smoking and alcohol consumption), family history, medical history, and reproductive history. According to their national origin, the participants were from 10 ethnic minorities, including Uygur ethnic minority, Chuang ethnic minority, Hui ethnic minority, Hmong ethnic minority, Tujia ethnic minority, Yi ethnic minority, Mongolian ethnic minority, Manchu ethnic minority, Tibetan ethnic minority, Buyi ethnic minority.

Venous whole blood samples were collected from participants by venipuncture. Hb level was measured with hematology analyzers immediately after blood sample collection in accordance with National Guide to Clinical Laboratory Procedures. The accuracy and stability of Hb measurements were ensured through the establishment of quality assurance system of the National Free Preconception Health Examination Project. The National Center of Clinical Laboratories for Quality Inspection and Detection did an external quality assessment (EQA) twice a year for quality control. The details were shown in other literature.^[7]^

Anemia was defined as Hb <120 g/L for nonpregnant women according to the WHO criteria, and divided into mild anemia and moderate-to-severe anemia with Hb level of 110 to 119 g/L and <110 g/L, respectively. Anemia was also classified by Chinese criteria (<110 g/L as anemia, 90–109 g/L as mild anemia, <90 g/L as moderate-to-severe anemia). Hb concentration of women in Tibetan was adjusted for altitude to eliminate the effect of altitude on prevalence of anemia according to the way from the WHO.^[8]^ The prevalence of anemia was normalized according to the composition of population from national census database in 2010.

### 1.3 Statistical analysis

Participants were primarily classified by ethnicity and proportions were used to describe the sociodemographic characteristics of age, education level, occupations aspects, and ethnic enclaves. Ethnic enclaves were divided according the government of China and details were as follows: Chuang's enclaves: Guangxi Chuang Autonomous Region; Uygur's enclaves: Xinjiang Uygur Autonomous Region; Hmong's enclaves: Guizhou Province, Hunan Province and Yunnan Province; Hui's enclaves: Ningxia Hui Autonomous Region; Tujia's enclaves: Guizhou Province, Hunan Province, Hunan Province and Chongqing Municipality; Yi's enclaves: Guizhou Province, Sichuan Province and Yunnan Province; Mongolian's enclaves: Inner Mongolia Autonomous Region; Manchu's enclaves: Hebei Province and Liaoning Province; Buyi's enclaves: Guizhou Province; Tibetan's enclaves: Qinghai Province and Tibet Autonomous Region. We used the mean and SD of the Hb concentration to estimate Hb level of each minority and adopted the multiple comparison of sample means (analysis of variance) to examine the differences of mean of Hb concentration among the 10 ethnic groups, as well as do Hb concentration between different ages, different education levels, different occupations of participants in the same ethnic group. We calculated the prevalence of anemia and its 95% confidenceinterval (CI) and the standardized rate of anemia were weighted by using the data of the sixth national population census. *χ*^2^ test was adopted to analyze the prevalence of anemia among the 10 ethnic groups. We considered 2-sided *P* values of <0.05 to be statistically significant. All analyses were performed with SPSS version 20.0 (SPSS Inc, Chicago, IL).

### 1.4 Role of the funding source

The sponsors had no role in study design, data collection, data analysis, data interpretation, and writing of the report. The corresponding author had access to all data sources and is responsible for the content of the report and had final responsibility for the decision to submit for publication.

## 2 Results

In the current study, 480,699 women were enrolled from 10 ethnic minorities. The participants were mostly 20 to 34 years’ old (89.4%), agriculture or livestock farming (88.3%), and less educated (83.4%). A summary of the demographic characteristics of study participants is shown in Table 1.

**Table 1:**
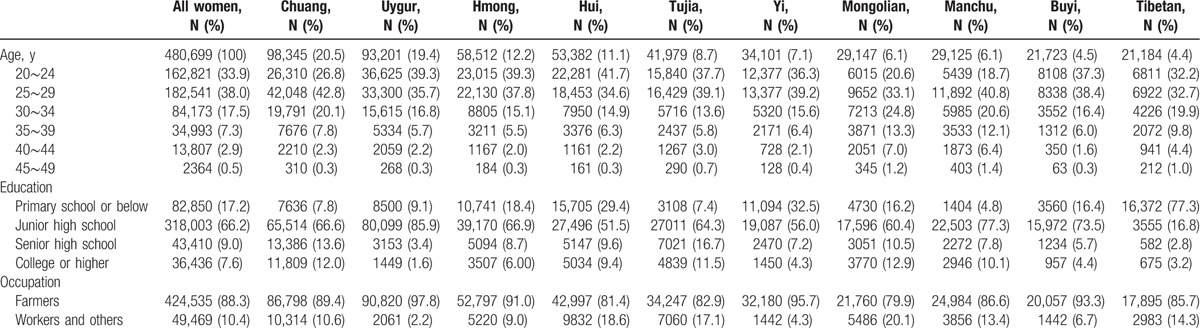
Characteristics of study participants.

The overall mean Hb concentration of all subjects was 131.2 g/L. The lowest mean Hb concentration was shown in Chuang (126.8 g/L), whereas the highest Hb concentration was in Tibetan (138.5 g/L) (Table 2). The Hb level of participants with primary school or below was higher than that in women with other education level in most ethnic minorities (Table 3).

**Table 2:**
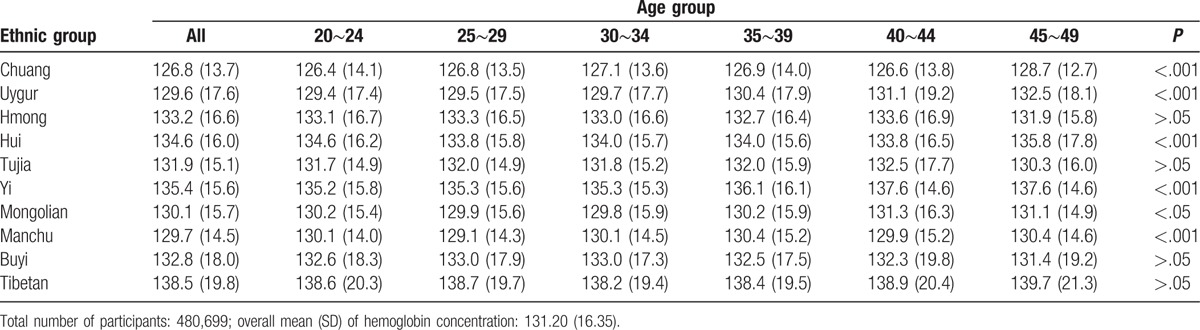
Comparison of mean hemoglobin concentration by age in different ethnic groups.

**Table 3:**
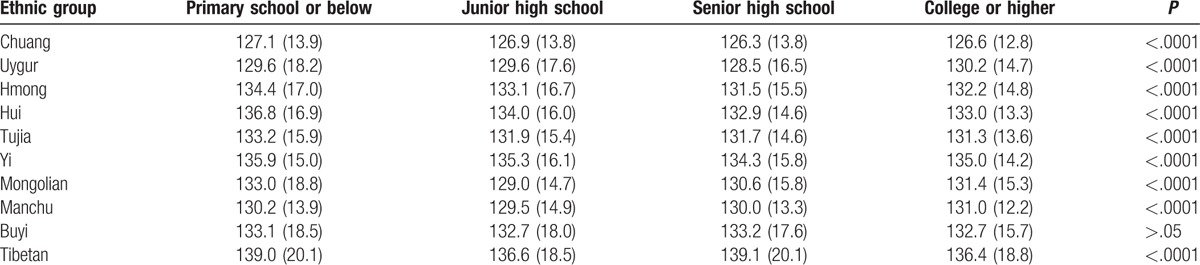
Comparison of mean hemoglobin concentration by education levels.

According to the WHO criteria to define anemia, the overall anemia rate was 21.3% in the 10 ethnic groups. The highest prevalence was in Tibetan (46.9%), and the lowest prevalence was in Yi (10.6%). The prevalence of anemia in each ethnic minority did not rise or decrease with age (Tables 4 and 5). After stratification according to the degree of anemia, the highest prevalence of mild anemia and moderate-to-severe anemia was also shown in Tibetan (17.9%, 28.9% respectively), and the lowest in Yi (8.4%, 3.6%). The mild anemia rate reduced with age in participants of most ethnic minorities, whereas the prevalence of moderate-to-severe anemia did not. The results of anemia rate were shown in Tables 6 and 7, when using Chinese criteria to define anemia.

**Table 4:**
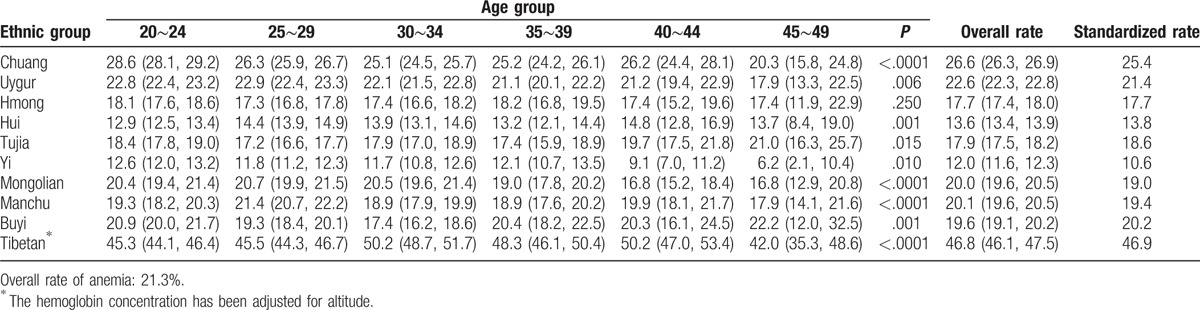
Prevalence of anemia by age in minorities (WHO criteria of anemia).

**Table 5:**
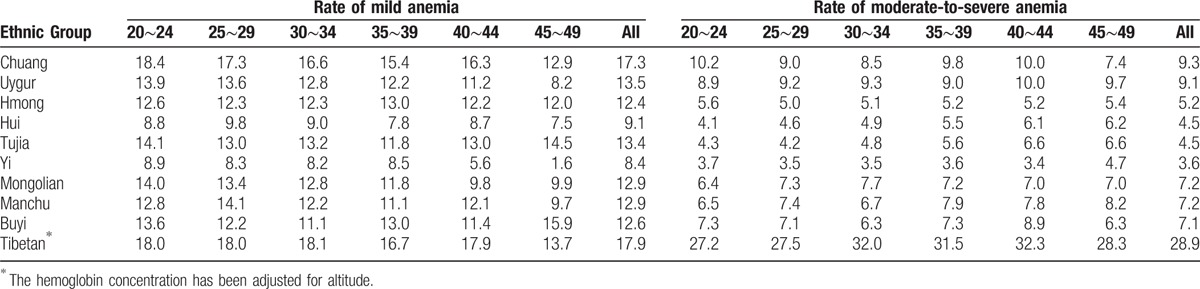
Prevalence of mild anemia by age in minorities (WHO criteria of anemia).

**Table 6:**
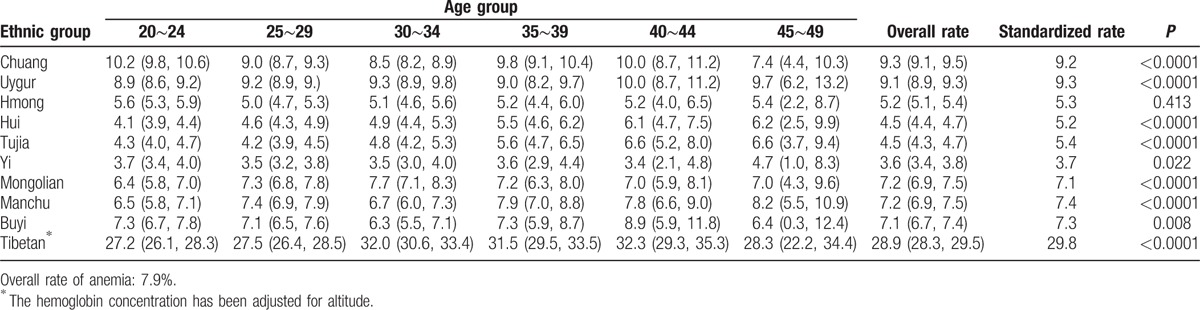
Prevalence of anemia by age in minorities (Chinese criteria of anemia).

**Table 7:**
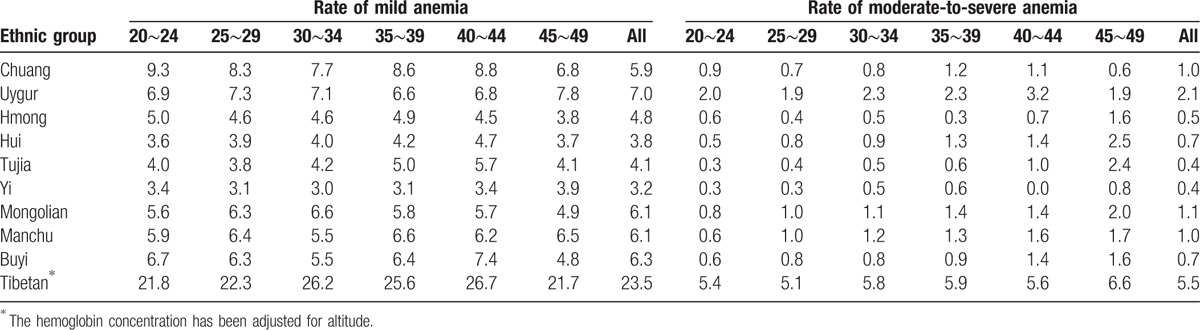
Prevalence of mild anemia by age in minorities (Chinese criteria of anemia).

Most participants (400412, 83%) lived in ethnic enclaves. The mean Hb concentrations were mostly higher in population from ethnic enclaves (Uygur, Hong, Hui, Tujia, Yi, and Buyi) than that from nonethnic enclaves. Consequently, the prevalence of anemia was lower in participants from ethnic enclaves (Uygur, Hong, Hui, Tujia, Yi, and Buyi) than that from nonethnic enclaves. Especially, the differences on mean Hb concentration or anemia rate in participants between ethnic enclaves and nonethnic enclaves were significant in Yi and Buyi (Table 8).

**Table 8:**
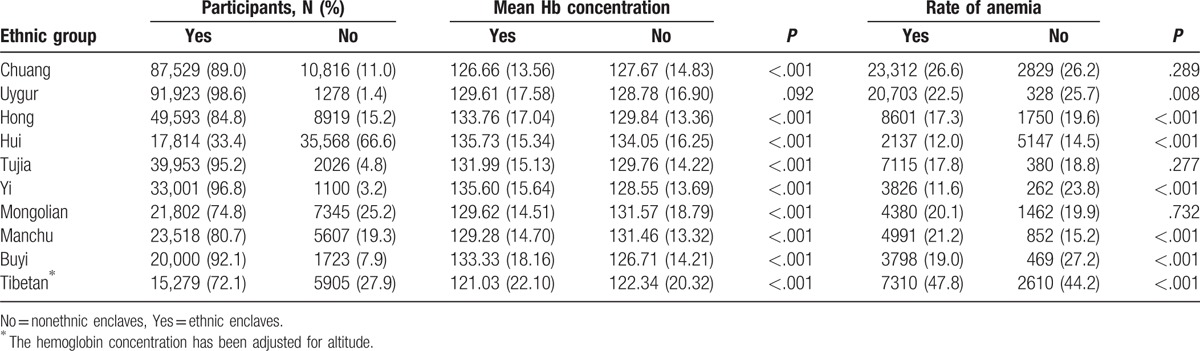
Distribution of hemoglobin and prevalence of anemia by ethnic enclaves.

## 3 Discussion

In the present study, ethnic differences of the Hb distributions and anemia prevalence were observed in the 10 ethnic groups. The lowest mean Hb concentration was shown in Chuang (126.8 g/L), and the highest mean Hb concentration was in Tibetan (138.5 g/L). After the adjustment of Hb concentration for altitude in Tibetan, the highest prevalence was in Tibetan (46.9%), and the lowest prevalence is in Yi (10.6%) when using WHO criteria to define anemia. Furthermore, there were differences on mean Hb concentration or anemia rate in participants between ethnic enclaves and nonethnic enclaves in most ethnic groups.

The overall mean Hb concentration of the 10 ethnic groups was 131.2 g/L, and was lower than the nationwide mean Hb concentration (133.45 g/L) of reproductive women reported from the China Nutrition and Health Survey in 2010–2012.^[4]^ The Hui's (134.6 g/L), the Yi's (135.4 g/L), and the Tibetan's (138.6 g/L) was higher, whereas the Chuang's (126.8 g/L), the Uygur's (129.6 g/L), the Manchu's (129.7 g/L), the Mongolian's (130.1 g/L), and the Tujia's (131.9 g/L) was significantly <133.45 g/L. This also suggested that there were ethnic differences of Hb distributions. Previous studies also showed that Hb distribution differed in races regardless of socioeconomic status.^[2,3]^ The possible causes are geographic conditions, diverse climate, human genetics, and eating habits. For example, the Tibetan mainly reside in plateau, as an adaptive response to reduced partial pressure of oxygen, Hb concentration in Tibetan increased significantly than that of other ethnic groups. For the Chuang where the rate of inherited hemoglobinpathies was high, the mean Hb concentration might be lower.^[9]^ Furthermore, the Manchu have the habit of drinking rice wine, and the Uygur like drinking tea. Whereas wine can reduce Hb level and tea rich in tannic acid affects absorption of iron.

In this study, there were significant differences of anemia rates among the 10 ethnic groups. The prevalence of anemia in Tibetan, Chuang, and Uygur was very high (46.9%, 25.4%, and 21.4% respectively), whereas the prevalence in Yi and Hui was very low, being 10.6% and 13.8%, respectively. The distribution of mild anemia or moderate-to-severe anemia is the same as above. The markedly high anemia rate of Tibetan might be associated with the hypoxic environment of the high-altitude Tibetan plateau. In addition, people of Tibetan have adapted to lower partial pressure of oxygen in the high altitude places and need not rely on increasing hemoglobin to supplement of oxygen.^[10]^ Therefore, the adjustment values for Tibetan might be biased if we adjust the Hb concentrations only for altitude and take on account of difference of genetic background of Tibetan that make us overestimate the rate of anemia of Tibetan ethnic minority. Studies showed that there was a high incidence of thalassemia in the Chuang; the frequency of α- and β-thalassemia carriers was 26.9% and 19.9%, respectively.^[9]^ Therefore, thalassemia should be the one of causes that the population of Chuang has high prevalence of anemia. For the Uygur, where the rate of inherited haemoglobinopathies was high, the prevalence of anemia might be increased.^[11]^ Anemia rate of Yi group (10.6%) was the lowest among the ten ethnic groups. The possible reason was the balance diet of the Yi including corn, beef and mutton, vegetables, and fruits which was beneficial to the absorption of iron and vitamins. The people of Hui are fond of beef and mutton, and they live in the areas with convenient transportation and high-level economic which make them less prone to develop iron-deficiency anemia.

The mean Hb concentration of Hong and Yi was significantly higher in ethnic enclaves than in nonethnic enclaves. The participants in Hmong enclaves liked pepper and tomatoes being rich in vitamin C, which could promote the absorption of iron in food,^[12]^ whereas those in nonethnic enclaves lost the traditional eating habits of Hmong. Similarly, the study showed that Yi's diet was rich in vitamin C and animal source foods, which lead to the apparent absorption rate of iron of Yi male adults was about 15%.^[13]^ Furthermore, the serum ferritin level affects the relationship between higher serum magnesium concentrations and lower risks of anemia.^[14]^ This might be the main reason for higher hemoglobin level and lower anemia rate in the participants of Yi enclaves. Similar reasons could explain the differences in mean Hb concentration and anemia rate between the participants of Buyi enclaves and those of non-Buyi enclaves. The women in Manchu enclaves mainly lived in Northeast of China where the climate is often cold and lacks fresh vegetables and fruits. They usually eat rice or wheat as staple food and like drinking. In contrast, the women of nonethnic enclaves mostly lived in economically developed areas and had a balanced diet. This was the possible reason for the difference in anemia rate between people of Manchu enclaves and those of nonethnic enclaves.

According to the criteria of anemia of WHO, the anemia rate of most ethnic groups was higher than the Nationwide prevalence of anemia (15.4%) in women of childbearing age in 2010 to 2012,^[4]^ which suggested that the status of ethnic anemia was still very serious, although the government has taken a series of measures to prevent anemia. Furthermore, the anemia rate of our participants except Tibetan's was higher than anemia rate in developed countries, such as American and Europe, 17.8% and 19.0% separately. In contrast, anemia rate of our participants except Tibetan's was lower than the worldwide anemia rate (30.2%) from the WHO Global Database in 1993 to 2005. The prevalence of anemia in the 10 ethnic groups was significantly lower than anemia rate in backward countries, such as in Africa and south-east Asia, the rate being 47.5% and 45.7%, respectively.^[1]^ However, according to Chinese anemia criteria, anemia rate of most ethnic groups in this study was far below the rates of developed countries. Causes of anemia are diverse, but in terms of public health, iron deficiency is so far the most important reason in the world. Physiological and pathological conditions can contribute to iron deficiency anemia. The absorption of iron from the diet is less than the body's requirement for iron, leading to a risk of iron deficiency. Women of childbearing age are especially at risk for iron-deficiency anemia on account of menstrual iron losses.^[15]^ Nutritional anemia also results from other causes, including a lack of folic acid, vitamin C, copper, and vitamin B12. In addition, thalassemia is determined by the defective gene.

A limitation of our study was that the program was designed for rural couple who plan to get pregnancy in 6 months, participants were mainly women aged 20 to 34 years, engaged in agriculture or animal husbandry, and had lower education level. Therefore, the data of each ethnic group could not completely represent its own Hb levels and status of anemia in reproductive women. The number of participants between ethnic enclaves and nonethnic enclaves was significantly different that might result in greater bias. The correlation analysis could not be done because of no data on the diet, socioeconomic, status and the rate of hemoglobinpathies.

In summary, ethnic differences of the Hb distribution and anemia prevalence were observed in the 10 ethnic groups which were associated with geographic conditions, diverse climate, genetic background, and eating habits.

## Acknowledgments

The authors thank health workers in 220 counties of 31 provinces for their strong collaboration and great effects made in the NFPHEP.
